# Editorial Notes, News and Miscellany

**Published:** 1913-05

**Authors:** 


### Editorial Notes, News and Miscellany.

A Chemical Tragedy.—Our Willie passed away today, his face we'll see no more; what Willie took for $H_2O$, proved $H_2SO_4$.

Dr. Geo. R. Tabor of Bryan, Texas, late State Health Officer of Texas, has entered the United States Public Health and Marine Hospital Service, and has been assigned to duty at Puerto, Mexico.

"The Surgical Solution of the Problem of Race Culture," by our associate of the Department of Eugenics, Dr. Malone Duggan, will appear in next issue. It was read to the general session of the State Association May 9, 1913.

An Exploded Myth.—Uncle Jack—I understand the angels brought you a little brother last night.

Small Bobby (pityingly)—You'd better come over to school tomorrow and join our class in sex hygiene.—*Lippincott's.*

The Supreme Court of Texas confirmed the finding of the lower court of Bexar County vs. Dr. White, for practicing medicine without a license. The fine is $500, in addition to this heavy fine, 90 days imprisonment was imposed. It comes high to violate the Medical Practice Act in Texas.

I have the pleasure of presenting my readers with a good photo of my associate editor, Mrs. D. She is also editor of the *Texas White Ribbon,* the official organ of the Women's Christian Temperance Union, and at the 1904 meeting of the Texas State Medical Association, was elected its "mascot."

Thought the Druggist Was Stingy.—An old servant stepped in and laid on the counter a prescription for a mixture containing two decigrammes of morphia. The chemist weighed the dangerous medicament with utmost care.

"What a shame!" then said the old woman, nudging his elbow. "Don't be so near; it is for an orphan girl!"

Before this reaches our readers, the big convention will have been held and passed into history. Of course we had to attend—Mrs. D. and I, and it came at the time when the journal should have gone to press, so that makes us late this time. I leave the report of the meeting to the Journal of the Association, that is its mission and *raison d'etre*, and as every member gets a copy, it would be superfluous in the "Red Back."

---

Modified Cow's Milk.—The Charles H. Phillips Chemical Company, New York, will send free, on request, Phillips' "Physician's Pocket Card for Home Modification of Cow's Milk," which gives full and explicit directions for this most important process. The attention of legislatures, as well as of health officers is now centered on the production, collection and distribution of cow's milk—the staff of life of the babies, and no doctor need be told that this attention is responsible for the great reduction of infant mortality in recent years. Drop a post card and get the card and show your patrons how to modify to suit the age of the babe. Mention the "Red Back."

---

T. Turner Thomas, of the University of Pennsylvania, in discussing local anesthesia for operations in the trigeminus region (Keen's *Surgery*, Vol. VI, p. 416) say: "The anesthetization of large nerve trunks by perineural injection of cocain solution was only possible or at least only practicable, on parts of the body on which the Esmarch constriction bandage could be applied. The addition of adrenalin to the cocain solution has made the method applicable to other parts of the body. The substitution of novocain for cocain permits the employment, without danger, of a much larger quantity of the anesthetic in the neighborhood of a nerve trunk, or to interrupt the conduction of a nerve for a much longer time.

---

Important if True.—It will be remembered that one of the Senators, in discussing (?) the sterilization bill, expressed the opinion that a cure would be found for idiocy, feeble-mindedness, imbecility and epilepsy, and that we should not deprive this class of their "God-given right to procreate." (See April "Red Back.") Report comes by wireless from New York that Flexner of the Rockefeller research laboratories has been to Texas and taken back with him a "culture" of senatorial blood, which he ran through a donkey —a Mexican burro—thereby "modifying" it, the result being a serum for the inoculation of future Senators against idiocy, feeble-mindedness and imbecility. We await with interest the report of the experiment.

Dr. J. A. Greene, of Blooming Grove, Texas, died at his home, April —, 1913. He was born in Union county, Mississippi, June 23, 1856; was educated in the common schools of the country, receiving a very good literary education; attended medical lectures at Louisville and at Mobile, graduating from the Mobile Medical College, March, 1881; attended a post-graduate course at Tulane in 1910, and specialized in diseases of the eye, ear, nose and throat. Returning to Blooming Grove, he was soon established in a good special practice. We have not many members of the profession like Dr. Greene, none to spare, and the journal counts his premature death a distinct loss to the profession of Texas. He never held any office in the Association. He was too modest even to sit in a front seat where he could be seen—and was—overlooked. D.

---

The Fourth International Congress on School Hygiene, and the first to be held in America, at Buffalo, August 25-30th, according to an announcement of the executive committee, will be by far the most elaborate effort yet made in this country toward getting the problem of school hygiene before the world. The first International Congress was held at Nuremberg in 1904, the second at London in 1907, the third at Paris in 1910.

The objects of the Buffalo Congress are:

(1) To bring together men and women interested in the health of school children.

(2) To organize a program of papers and discussions covering the field of school hygiene.

(3) To assemble a school exhibit representing the best that is being done in school hygiene.

(4) To secure a commercial exhibit of practical and educational value to school people.

(5) To publish the proceedings of this Congress and distribute them to each member.

In addition there is a plan on foot to effect a permanent organization for the purpose of carrying out school hygiene reforms in all the individual communities in this country, if not all over the world.

One of the interesting features of the Congress will be the presence of delegates representing the community interest in school hygiene, including those appointed by mayors and governors, by women's clubs, by school boards, boards of health, by mothers' congresses and charity organization societies and boards of trade. Their help is being solicited with a view of organizing the community in a campaign of school hygiene reform. * * *

President Wilson has accepted the honorary office of patron of the Congress. The president of the Congress is Mr. Charles W. Eliot of Harvard University. The vice-presidents are Dr. William H. Welch, of Johns Hopkins University, and Dr. Henry P. Walcott, president of the recent International Congress on School Hygiene and Demography, and chairman of the Massachusetts State Board of Health.

For full program, address C. S. Thompson, College of the City of New York.

